# Muech-Weiss Tubercle Granules

**Published:** 1917-04

**Authors:** 


**Muech-Weiss Tubercle Granules.** Edit. Review in Jour. of Lab. & Clin. Med., Feb. Meader thinks they may be degenerate tubercle bacilli, having lost their acid-fast character by absence of the wax coating of the bacillus. Fishberg considers them diagnostic of tuberculosis in old lesions as cold abscesses. On the other hand, apparent demonstration of their nature by the development of tuberculosis in guinea pigs, should be discounted as less than 10 tubercle bacilli, which might easily be overlooked, would be adequate to cause infection. The editor states that he has failed to produce tuberculosis by the injection of sputum containing Muech-Weiss granules without bacilli and, on the other hand, has succeeded with sputum in which neither could be found microscopically. A case in a man of 60, clinically diagnosed as tuberculosis, typic tubercle bacilli never found but granules abundant, came to necropsy and no evidence of tuberculosis could be found but, instead, chronic interstitial pneumonia. Fraenkel and Muech have found these granules in the lymph glands in Hodgkins Disease and lymphatic leukaemia. As the demonstration of the granules requires the concentration of two or three ounces of sputum and is laborious, it is not recommended for clinical purposes unless further evidence of its diagnostic value is adduced.

